# Self-rated oral health status, oral health service utilization, and oral hygiene practices among adult Nigerians

**DOI:** 10.1186/1472-6831-14-140

**Published:** 2014-11-27

**Authors:** Adeyemi Oluniyi Olusile, Abiola Adetokunbo Adeniyi, Olufemi Orebanjo

**Affiliations:** Department of Restorative Dentistry, Faculty of Dentistry, Obafemi Awolowo University, Ile-Ife, Osun State Nigeria; Department of Preventive Dentistry, Faculty of Dentistry, Lagos State University College of Medicine, Lagos, Nigeria; Dental Department, Ajeromi Ifelodun General Hospital, Lagos, Nigeria

**Keywords:** Socio-demographic factors, Oral health, Oral hygiene, Toothbrushing, Dental attendance, Nigerians

## Abstract

**Background:**

There is scarce information available on oral health service utilization patterns and common oral hygiene practices among adult Nigerians. We conducted the 2010–2011 national oral health survey before the introduction of the national oral health policy to determine the prevalence of oral health service utilization, patterns of oral hygiene practices, and self reported oral health status, among adults in various social classes, educational strata, ethnic groups and geopolitical zones in Nigeria.

**Methods:**

We conducted a cross-sectional survey in North-Central, North-West, South-East, South-South and South-West geopolitical zones of Nigeria. Multi-stage cluster sampling method was used for the sample selection. We administered a structured questionnaire to a total of 7,630 participants. Information on the socio-demographic characteristics, oral hygiene practices and oral health services utilization pattern of participants was obtained.

**Results:**

We interviewed 7, 630 participants (55.6% female). The participants ages ranged between 18 and 81 years, mean age was 37.96 (SD = 13.2). Overall 21.2% of the participants rated their oral health status as very good, 37.1% as good and 27.4% as fair. Only 26.4% reported having visited the dentist at least once prior to the conduct of the survey. More than half of these visits (54.9%) were for treatment purpose. Utilization of oral health services was significantly (p < 0.05) associated with being older, more educated and being engaged in a skilled profession. More educated persons, females and younger persons used toothbrushes for daily tooth cleaning. Age, sex, marital status, level of education and occupation were significantly related to daily frequency of tooth cleaning (p < 0.05).

**Conclusion:**

Our results show that while most Nigerian adults have a positive view of their oral health status, majority reported poor oral health utilization habits. Older persons resident in the northern zones of the country and less educated persons displayed poorer oral hygiene practices. The study findings suggest that there is low oral health service utilization among adult Nigerians and that socio-demographic variables influence oral health utilization habits and oral hygiene behavior among adult Nigerians Further studies to identify other factors influencing oral health behavior are suggested.

## Background

Oral health is an important tool for achieving good general health [1]. Oral diseases and disorders often result in physical discomfort, pain, infection and sometimes tooth loss [2]. They also frequently cause difficulty in chewing; swallowing; speaking, and can disrupt sleep and productivity [1, 2]. Consequently, oral diseases and disorders not only affect the victim’s life as well as their social networks, the community at large and productivity of the citizens at the national level [2–4].

The promotion of good oral hygiene at the population level is advocated and supported by the World Health Organisation (WHO) and the International Federation of Dentists (FDI) [5–8]. The adoption of preventive strategies both at the individual and population level helps reduce the negative impact of oral diseases including improving quality of life. One critical tool identified for achieving good oral health is the institution of effective and efficient oral hygiene practices [1, 5, 9]. The value of good oral hygiene practices has increased over the years.

Research indicates that the removal of bacteria plaque is essential for the prevention of the two most prevalent dental conditions namely dental caries and periodontal disease [5, 9]. To achieve and maintain good oral hygiene, and prevent dental caries regular tooth brushing using fluoride containing toothpaste at least twice a day is recommended. [8] The use of dental floss for cleaning of interproximal surfaces is also crucial for effective plaque removal [7, 8].

In Nigeria, information on oral health service utilization patterns and common oral hygiene practices among adult Nigerians is sparse. Most of the available information is from research localized to south west Nigeria or to specific sub-populations such as pregnant women and school going children [10–12]. Research reports have consistently confirmed that a large proportion (more than 70%) of adult Nigerians have periodontal disease [11, 13], a condition strongly associated with oral hygiene status. Although in the last two decades reports using the decayed, missing and filled teeth (DMFT) index, show a low dental caries prevalence of 10% to 20%, dental caries remain a public health concern because most carious lesions remain untreated [10–13]. Reports also indicate poor awareness of oral health, irregular tooth brushing and generally poor oral hygiene among Nigerians [10, 13]. Other data indicates that very few Nigerians (less than 20%) visit a dentist regularly [10–12]. This may be related to the inadequate number and poor geographical distribution of oral health care providers countrywide; factors which do not favor adequate access to oral health care services [10, 12].

Elsewhere evidence shows that oral health care utilization patterns and oral hygiene habits are influenced by socio-demographic characteristics [2, 3]. Persons with higher education and a higher social economic status have been reported to exhibit better oral hygiene habits and oral health utilization habits [2, 3, 14–16]. However, the effect of socio-demographic characteristics on oral health service utilization in Nigeria is not documented. To our knowledge this is the first national survey conducted to assess the oral health service utilization pattern and oral hygiene practices of adult Nigerians.

This survey was conducted before the introduction of the national oral health policy in Nigeria [12] with an overall goal to provide veritable information for the implementation, monitoring and evaluation of the proposed national oral health policy. The study also aimed at determining the association between socioeconomic status, educational status, ethnic groups and geopolitical zones in Nigeria with the prevalence of oral health service utilization and pattern of oral hygiene practices among adults in Nigeria.

## Methods

### Study design

The study was carried out between Jan 2010 and June 2011. The study was population based and using a rapid assessment survey method. This approach was adopted because there was limited time and resources for collecting the data before the introduction of the national oral health policy. The survey tool was a questionnaire to assess the oral health service utilization and pattern of oral hygiene practices of participants. The survey tool was administered in person and had a target length of fifteen minutes. It was developed and refined by the authors with input from Dentists in other parts of the country. It was designed for ease of use with minimal interviewer training.

### Study population

The estimated total population of Nigeria at the time of conducting this study was 159 million, out of which 54.94% are aged 18 to 64 years while 3% are aged 65 years and older [17]. The target population for this survey was approximately 92 million persons aged 18 years and older.

### Study location

The study was conducted in Nigeria, which comprises 36 states and the federal capital territory. The states have been categorised into six geo-political zones or regions for ease of administration namely North-Central, North-East, North-West, South-East, South-South and South-West. Each state is further divided into three senatorial districts and each senatorial district divided into local Government areas (LGA). There are 774 LGA’s in Nigeria altogether. The LGA is the smallest unit of administration in the country.

### Sampling method

We used a multi-stage cluster sampling method to conduct this survey. Five states in the north-eastern geo-political zone that were experiencing socio-political problems (namely Adamawa, Zamfara, Plateau, Borno, and Yobe) during the data collection period were excluded from the sampling frame. Eighteen out of the remaining 31 States were randomly selected for the study. The selected states included Abia, Anambra, Bayelsa, Benue, Ekiti, Enugu, Imo, Kaduna, Kano, Kogi, Kwara, Lagos, Nasarawa, Niger, Ogun, Ondo, Osun and Oyo. One LGA per three senatorial districts in each of the 18 states was randomly selected using the list of LGA’s in the state as the sampling frame. In total, 54 LGA’s were visited during data collection.

### Sample selection

The research assistants were dentists resident in each of the selected states. They regularly met with the health care team in the selected LGA to obtain permission to conduct the study. Before the visit, community leaders were informed of the proposed study, their consent sought and they were encouraged to mobilise participants from their locality for the study. Participants were sequentially recruited based on the following criteria: must be an 18 years or older, should reside in the LGA and be willing to participate in the research. Subjects 18 years and below or who were unwilling to participate were excluded from the study.

### Data collection

Data collection was done in a central location in the community (local government headquarters or town hall). Data on demographic characteristics, oral health service utilisation, patterns of oral hygiene practices, and self-reported oral health status were collected using a structured questionnaire. The occupation was recoded for data entry using the UK registrar’s occupation classification [18] while age was recoded using the decades of life. The second section asked questions on oral hygiene practices and oral health services utilization patterns. Under oral hygiene practices, we specifically enquired about the tools used for cleaning the mouth, frequency of daily mouth cleaning, use of adjunct tools such as dental floss and mouthwash and the estimated duration of mouth cleaning. Under oral health service utilization, we enquired about history of previous visits to oral health care units and reasons for those visits.

Literate participants completed the questionnaire personally while trained interviewers assisted illiterate subjects using pidgin English (a local form of English in the country) or one of the three main languages in the country. The survey instrument was translated and back translated to pidgin and three other major languages including Yoruba, Ibo and Hausa. The translated versions were used during the training and standardization of the interviewers. Interviewers used the translated versions strictly for illiterate persons. The questionnaire was pretested to ensure simplicity and ease of understanding by the participants. Changes were made to the questionnaire before data collection.

### Ethical considerations

Ethical clearance for the study was obtained from the Ethical Review Committee of the College of Health Sciences Obafemi Awolowo University Ile-Ife Nigeria. In addition, permissions to conduct the study were also obtained from the chairpersons of all the selected LGAs, through the directors of primary health care. The researchers explained to all study participants the scope, aims and objectives of the study as well as their rights to participate or withdraw from the study with no penalty. All study participants gave verbal consent and were assured that their confidentiality would be maintained.

### Data analysis

Data entry and analysis was done using SPSS statistical software version 16.0. Univariate analysis was carried out; means, standard deviation were computed for quantitative variables and frequency distributions generated for qualitative variables. Bivariate analysis to identify associations between oral hygiene habits (i.e. the dependent variables namely frequency of tooth brushing, tool used and duration of toothbrushing) and sex, age, geo-political zone, and educational status (the independent variables) of the study participants was also carried out. The chi-squared test was used as test of significance for comparing proportions for more than 2 groups while the fishers exact was used for comparing differences in proportion between two groups. The student t-test was used to compare differences in age. Logistic regression analysis was conducted to identify factors independently associated with adequate tooth brushing frequency in the population (defined as brushing two or more times daily). Associations were considered significant when the p-values were equal or less than 0.05.

## Results

A total of 7,630 persons aged 18 to 81 years comprising 44.4% men and 55.6% women participated in the survey. The mean age (SD) of the participants was 37.96 (13.2) years while majority of the participants (62.3%) were married. Table 1 summarizes the characteristics of the study population by gender. Males were significantly older (more than 50% of persons aged 50 years and above were male). More males than females participated in the northern part of the country (66.2% in North west and 46.1% in North Central).

**Table 1:** **Showing socio-demographic characteristics of participants by gender**

Social characteristic	Female		Male		Total		Χ^2^
							***df***
							(p)
	No	%	No	%	No	%	
**Geo-political zone**							
North-Central	1003	53.9	859	46.1	1862	24.4	317.38
North-West	401	33.8	787	66.2	1188	15.6	*4*
South-East	1074	64.1	602	35.9	1676	22.0	(<0.001)
South-South	231	56.2	180	43.8	411	5.4	
South-West	1536	61.6	957	38.4	2493	32.6	
**Age category**							
<20 years	254	53.1	224	46.9	478	6.3	51.01
20–29 years	1012	58.4	721	41.6	1733	22.7	*5*
30–39 years	1209	59.5	822	40.5	2031	26.6	(<0.001)
40–49 years	1018	55.4	820	44.6	1838	24.1	
50 – 59 years	484	48.8	507	51.2	991	13.0	
60 years and above	268	47.9	291	52.1	559	7.3	
**Educational Status**							
Unspecified	536	56.7	410	43.3	946	12.4	33.03
None	422	60.5	276	39.5	698	9.1	*4*
Primary school	424	49.2	438	50.8	862	11.3	(<0.001)
Secondary school	1086	53.0	963	47.0	2049	26.9	
Tertiary institution	1777	57.8	1298	42.2	3075	40.3	
**Marital Status**							
Unspecified	450	52.2	412	47.8	862	11.3	169.74
Single	864	53.0	767	47.0	1631	21.4	*4*
Married	2604	54.7	2153	45.3	4757	62.3	(<0.001)
Separated	44	63.8	25	36.2	69	0.9	
Widow(er)	283	91.0	28	9.0	311	4.1	
**Occupation**							
None	797	59.8	537	40.2	1332	17.5	14.35
Student	296	57.1	222	42.9	518	6.8	*4*
Unskilled worker	1059	55.0	866	45.0	1925	25.2	(0.006)
Skilled worker	1167	53.4	1018	46.6	2185	28.6	
Very skilled worker	926	55.4	744	44.6	1670	21.9	
**Total**	**4245**	**55.6**	**3385**	**44.4**	**7630**	**100.0**	

### Perception of oral health status and oral health service utilization patterns

Overall 21.2% of the participants rated their oral health status as very good, 37.1% as good, 27.4% as fair, 9.0% as poor or very poor while the remaining were not sure of their oral health status. Only 26.4% reported having visited the dentist prior to the conduct of this survey. More than half of these visits (54.9%) were for treatment, 24.9% were for check-up only and the remaining participants reported visiting for both treatment and check-up.

### Oral hygiene practices

The oral hygiene tool used by the largest proportion of participants was the toothbrush and toothpaste (81% of participants). Other tools used included chewing stick (9.6%), salt (0.6%), water only (0.5%) and cotton wool (0.3%). Some participants (5.6%) reported using multiple tools. The commonest combination used was the toothbrush and chewing stick (81.4% of those who used multiple tools).

About 20% of the participants reported using fluoride-containing toothpastes, 10.5% used toothpastes without fluoride while the remaining (69.6%) was unsure of the fluoride content of their dentifrice. Only 10.5% of the participants reported using dental floss or other oral hygiene aids such as mouthwashes. Also, 42.0% of participants reported cleaning their mouth twice daily while 37.1% clean their mouth once a day. Participants also reported on the amount of time spent cleaning their mouths: 24.7% spend 1–2 minutes, 34.4% spend between 3 and 4 minutes, 25.9% spend more than 5 minutes while the remaining were uncertain.

### Association between socio-demographic features, oral health service utilization and oral hygiene practices

Persons in older age categories (i.e. 50 years and older) reported having visited the dentist before this study was conducted significantly more than those in the younger age categories (p < 0.001). Similarly, persons who had more education i.e. tertiary education (p < 0.001) as well as persons in more skilled professions (p < 0.001) reported significantly more utilization of oral health services (Table 2). Sex and geopolitical zoning were not significantly associated with previous utilization of oral health services. The use of toothbrush was significantly related to age (p < 0.001), sex (p < 0.001), educational status (p < 0.001), and occupation (p < 0.001). Younger persons (p < 0.001), females (p < 0.001) and more educated persons (p < 0.001) used toothbrushes for daily tooth cleaning. Similarly all the socio-demographic characteristics were significantly related (p < 0.001) to daily frequency of tooth cleaning (Table 3). Table 4 displays the relationship between duration of mouth cleaning and socio demographic characteristics. Sex was not significantly related to the duration of tooth cleaning. The relationship between duration of tooth cleaning and the tool used was significant (p < 0.001), persons using toothbrush reported significantly less tooth cleaning time than those using other tools.

**Table 2:** **Showing the relationship between socio-demographic features, dental attendance and oral hygiene tools used**

	Have you ever been to a dentist?		Oral hygiene tools used		Total (%)
Socio-demographic variable	Yes (%)	No (%)	Toothbrush (%)	Other tools (%)	
**Age category**					
<20 years	86 (18.0)	392 (82.0)	440 (92.1)	38 (7.9)	478 (6.3)
20–29 years	352 (20.3)	1381 (79.7)	1520 (87.7)	213 (12.3)	1733 (22.7)
30–39 years	512 (25.2)	1519 (74.8)	1680 (82.7)	351 (17.3)	2031 (26.6)
40–49 years	559 (30.4)	1279 (69.6)	1454 (79.1)	384 (20.9)	1838 (24.1)
50 – 59 years	338 (34.1)	653 (65.9)	733 (74.0)	258 (26.0)	991 (13.0)
60 years and above	169 (30.2)	390 (69.8)	336 (60.1)	223 (39.9)	559 (7.3)
	Χ^2^ = 101.64 p < 0.001		Χ^2^ = 284.35 p < 0.001		
**Gender**					
Female	1144 (26.9)	3161 (73.1)	3582 (84.4)	663 (15.6)	4245 (55.6)
Male	872 (25.8)	2513 (74.2)	2581 (76.2)	804 (23.8)	3385 (44.4)
	p = 0.250		p < 0.001		
**Educational Status**					
Unspecified	226 (23.9)	720 (76.1)	736 (77.8)	210 (22.2)	946 (12.4)
None	133 (19.1)	565 (80.9)	424 (60.7)	274 (39.3)	698 (9.1)
Primary school	219 (25.4)	643 (74.6)	587 (68.1)	275 (31.9)	862 (11.3)
Secondary school	467 (22.8)	1582 (77.2)	1674 (81.7)	375 (18.3)	2049 (26.9)
Tertiary institution	971 (31.6)	2104 (68.4)	2742 (89.2)	333 (10.8)	3075 (40.3)
	Χ^2^ = 78.99 p < 0.001		Χ^2^ = 415.61 p < 0.001		
**Occupation**					
Unspecified	317 (23.8)	1015 (76.2)	1028 (77.2)	304 (22.8)	1332 (17.5)
Student	90 (17.4)	428 (82.6)	463 (89.4)	55 (10.6)	518 (6.8)
Unskilled worker	432 (22.4)	1493 (77.6)	1355 (70.4)	570 (29.6)	1925 (25.2)
Skilled worker	622 (28.5)	1563 (71.5)	1851 (84.7)	334 (15.3)	2185 (28.6)
Very skilled worker	555 (33.2)	1115 (66.8)	1466 (87.8)	204 (12.2)	1670 (21.9)
	Χ^2^ = 86.77 p < 0.001		Χ^2^ = 244.17 p < 0.001		
**Geo-political Zone**					
North-Central	488 (26.2)	1374 (73.8)	1652 (88.7)	210 (11.3)	1862 (24.4)
North-West	336 (28.3)	852 (71.7)	775 (65.2)	413 (34.8)	1188 (15.6)
South-East	459 (27.4)	1217 (72.6)	1302 (77.7)	374 (22.3)	1676 (22.0)
South-South	108 (26.3)	303 (73.7)	352 (85.6)	59 (14.4)	411 (5.4)
South-West	625 (25.1)	1868 (74.9)	2082 (83.5)	411 (16.5)	249 (32.7)
	Χ^2^ = 53.1 p = 0.257		Χ^2^ = 289.06 p < 0.001		
**Total**	**2016 (26.4)**	**5614 (73.6)**	**6163 (80.8)**	**1467(19.2)**	**7630 (100.0)**

**Table 3:** **Showing the relationship between socio-demographic features and daily frequency of tooth cleaning**

Socio-demographic variable	Daily frequency of tooth cleaning					Total (%)
	Varies (%)	Not daily (%)	Once (%)	Twice (%)	More than twice (%)	
**Age category**						
<20 years	74 (15.5)	3 (0.6)	131 (27.4)	234 (49.0)	36 (7.5)	478 (6.3)
20–29 years	277 (16.0)	21 (1.2)	546 (31.5)	786 (45.4)	103 (5.9)	1733 (22.7)
30–39 years	306 (15.0)	16 (0.8)	770 (37.9)	830 (40.9)	111 (5.5)	2031 (26.6)
40–49 years	220 (12.0)	20 (1.1)	696 (37.9)	824 (44.8)	78 (4.2)	1838 (24.1)
50 – 59 years	123 (12.4)	15 (1.5)	435 (43.9)	346 (34.9)	72 (7.3)	991 (13.0)
60 years and above	57 (10.2)	10 (1.8)	254 (45.4)	187 (33.5)	51 (9.1)	559 (7.3)
	Χ^2^ = 137.13 df = 20 p < 0.001					
**Gender**						
Female	569 (13.4)	23 (0.5)	1550 (36.5)	1906 (45.0)	197 (4.6)	4245 (55.6)
Male	486 (14.4)	62 (1.8)	1282 (37.9)	1301 (38.4)	254 (7.5)	3385 (44.4)
	Χ^2^ = 75.14 df = 4 p < 0.001					
**Educational Status**						
Unspecified	44 (4.7)	11 (1.2)	285 (30.1)	399 (42.2)	44 (4.7)	946 (12.4)
None	52 (7.4)	23 (3.3)	270 (38.7)	245 (35.1)	52 (7.4)	698 (9.1)
Primary school	51 (5.9)	9 (1.0)	363 (42.1)	307 (35.6)	51 (5.9)	862 (11.3)
Secondary school	143 (7.4)	28 (1.4)	701 (34.2)	896 (43.7)	143 (7.0)	2049 (26.9)
Tertiary institution	161 (5.2)	14 (0.5)	1213 (39.4)	1360 (44.2)	161 (5.2)	3075 (40.3)
	Χ^2^ = 173.72 df = 16 p < 0.001					
**Occupation**						
Unspecified	274 (20.60	13 (1.0)	439 (33.0)	530 (39.8)	76 (5.7)	1332 (17.5)
Student	98 (18.9)	2 (0.4)	159 (30.7)	233 (45.0)	26 (5.0)	518 (6.8)
Unskilled worker	301 (15.6)	43 (2.2)	713 (37.0)	753 (39.1)	113 (6.0)	1925 (25.2)
Skilled worker	314 (14.4)	17 (0.8)	804 (36.8)	901 (41.2)	149 (6.8)	2185 (28.6)
Very skilled worker	68 (4.1)	10 (0.6)	717 (42.9)	790 (47.3)	85 (5.1)	1670 (21.9)
	Χ^2^ = 254.95 df = 16 p < 0.001					
**Geo-political Zone**						
North-Central	306 (16.4)	13 (0.7)	617 (33.1)	832 (44.7)	94 (5.0)	1862 (24.4)
North-West	258 (21.7)	36 (3.0)	305(25.7)	483 (40.7)	106 (8.9)	1188 (15.6)
South-East	73 (4.4)	24 (1.4)	666 (39.7)	784 (46.8)	129 (7.7)	1676 (22.0)
South-South	13 (3.2)	8 (1.9)	172 (41.8)	195 (47.4)	23 (5.6)	411 (5.4)
South-West	405 (16.2)	4 (0.2)	1072 (43.0)	913 (36.6)	99 (4.0)	2493 (32.7)
	Χ^2^ = 439.3 df = 16 p < 0.001					
**TOTAL**	**1055 (13.8)**	**85 (1.1)**	**2832 (37.1)**	**3207 (42.1)**	**451 (5.9)**	**7630 (100.0)**

**Table 4:** **Showing the relationship between socio-demographic features and duration of tooth cleaning**

	Duration of tooth cleaning				
Socio-demographic variable	Less than 2 minutes (%)	More than 2 minutes (%)	More than 5 minutes (%)	Not sure (%)	Total (%)
**Age category**					
<20 years	105 (22.0)	142 (29.7)	117 (24.5)	114 (23.8)	478 (6.3)
20–29 years	413 (23.8)	597 (34.4)	434 (25.0)	289 (16.7)	1733 (22.7)
30–39 years	508 (25.0)	765 (37.2)	450 (22.2)	317 (15.6)	2013 (26.6)
40–49 years	440 (23.9)	649 (35.3)	506 (27.5)	243 (13.2)	1838 (24.1)
50 – 59 years	290 (29.3)	304 (30.7)	273 (27.5)	124 (12.50	991 (13.0)
60 years and above	126 922.5)	175 (31.3)	198 (35.4)	60 (10.7)	559 (7.3)
	Χ^2^ = 104.88 df = 15 p < 0.001				
**Gender**					
Female	1005 (23.7)	1505 (35.5)	1111(26.2)	624 (14.7)	4245 (55.6)
Male	877 (25.9)	1118(33.0)	867 (25.6)	523 (15.5)	3385 (44.4)
	Χ^2^ = 8.196 df = 4 p = 0.085				
**Educational Status**					
Missing	192 (20.3)	223 (23.6)	194 (20.5)	337 (35.6)	946 (12.4)
None	194 (27.8)	155 (22.2)	230 (33.0)	119 (17.0)	698 (9.1)
Primary school	237 (27.5)	223 (27.0)	264 (30.6)	128 (14.8)	862 (11.3)
Secondary school	497 (24.3)	688 (33.5)	595 (29.0)	269 (13.1)	2049 (26.9)
Tertiary institution	762 (24.8)	1324 (43.0)	695 (22.6)	294 (9.6)	3075 940.3)
	Χ^2^ = 548.21 df = 12 p < 0.001				
**Occupation**					
None	327 (24.50	360 (27.1)	297 (22.3)	348 (26.1)	1332 (17.5)
Student	118 (22.8)	190 (36.7)	113(21.8)	97 (18.7)	518 (6.8)
Unskilled worker	496 925.8)	517 (26.9)	584 (30.3)	328 (17.0)	1925 (25.2)
Skilled worker	529 (24.2)	803 (36.8)	562 (25.7)	297 (13.3)	2185 (28.6)
Very skilled worker	412 (24.7)	753 (45.1)	422 (25.3)	83 (5.0)	1670 (21.9)
	Χ^2^ = 675.114 df = 12 p < 0.001				
**Geo-political Zone**					
North-Central	473 (25.4)	595 (32.0)	453 (24.3)	341 (18.3)	1862 (24.4)
North-West	297 925.0)	193 (16.3)	226 (19.0)	472 (39.7)	1188 (15.6)
South-East	477 (28.5)	652 (38.9)	437 (26.1)	110 (6.6)	1676 (22.0)
South-South	151 (36.7)	149 (36.3)	75 (18.2)	36 (8.8)	411 (5.4)
South-West	484 (19.4)	1034 (41.5)	787 (31.6)	188 (7.5)	2493 (32.7)
	Χ^2^ = 968.99 df = 12 p < 0.001				
**TOTAL**	**1882 (24.7)**	**2623 (34.4)**	**1978 (25.9)**	**1147 (15.0)**	**7630 (100.0)**

Regression analysis indicates that the relationship between the socio-demographic characteristics of the study population and their frequency of tooth cleaning was significant (p <0.001). Persons with 12 or more years of education were 15.7% more likely to clean their teeth at least twice daily; those from the southern part of the country were 5.2% more likely to brush at least twice daily. With every year increase in age there was 1.7% reduction in the likelihood of twice daily cleaning. While a previous dental visit or exposure to oral health education also increased the likelihood of twice-daily tooth cleaning by 42.3% (Table 5).

**Table 5:** **Regression analysis of factors determining frequency of toothbrushing**

Variable	Frequency of tooth cleaning			
	***Odds ratio***	***Lower CI***	***Upper CI***	***p-value***
**Age**	-0.017	-0.976	0.990	<0.001
**Gender**	-0.322	0.605	0.868	<0.001
**Geo-political zone (North Vs South)**	0.052	0.863	1.286	0.609
**Educational status**	0.157	1.034	1.324	0.013
**Previous visit to the dentist**	0.423	1.261	1.847	<0.001
**Previous oral health education**	0.300	1.113	1.638	0.002

## Discussion

This is the first national study to be conducted on oral health care service utilization and oral hygiene practices among adult Nigerians resident in the different geopolitical regions. Most of the earlier studies have been localized to specific regions in the country or have targeted specific populations such as pregnant women or school children [19–22]. Our results reveal that adult Nigerians view their oral health status positively but have poor regular oral health utilization habits and oral hygiene habits. A good number of the participants do not clean twice their teeth daily (52.0%) or brush for at least two minutes each time (39.7%).

A good proportion (58.3%) of the study participants viewed their oral health status as good or very good. This perception may explain the apathy displayed towards oral health service utilization as just over a quarter of the participants had ever visited a dentist. Anecdotal reports suggest that Nigerians generally associate not visiting a health facility with good health and similarly not visiting a dentist with good oral health. Therefore, it is common to visit the dentist only when they have symptoms that have not responded to home therapies. Furthermore, there is a tendency for adults to initially seek the attention of alternative health care practitioners before visiting a health facility. This in our view may explain the low utilization rates recorded. However, it is worth noting that the fact that oral health services are not readily available in all communities may limit the utilization of oral health services by most Nigerians [10–12].

We recorded a higher percentage (26.4%) of previous dental visits in this study than has been reported previously [23]. Differences in the results obtained may be because the earlier study was conducted at household level and was restricted to one town in south west Nigeria. However, oral health service utilization rates in our study were lower than previously reported in other countries [24–26]. A pattern of not visiting the dentist regularly has been reported among various groups in Nigeria [11–13, 18, 19, 21, 22] indicating a need to actively promote the utilization of dental services in the country as part of a strategy for achieving good overall health. One suggested approach for achieving increased utilization of dental services is the institution of national guidelines regarding dental visits e.g. integration of oral health into other essential medical services such as antenatal and postnatal care, as well as services for medically compromised persons and the elderly.

We also observed that age group, educational status and occupational status were associated with utilization of dental services, a finding reported in other countries [27, 28]. The fact that older persons reported significantly more previous dental visits than younger persons was not surprising. Older persons tend to have more dental problems [2, 27, 28], since most of the reported visits were sequel to the onset of dental symptoms it can inferred that the visits were made by older persons. As expected, more educated persons and more skilled workers also reported higher prevalence of previous dental visits than those with less education and those in less skilled professions. However gender and geo-political zone were not related to previous dental visits.

Majority of the participants reported using toothbrushes for cleaning. Generally, a higher proportion of older persons utilized other tools especially the chewing stick for cleaning. This finding may be an indication of a shift towards the use of modern tools such as the toothbrush among Nigerian adults. Persons in the younger age groups may have been influenced by information obtained from school and the media as they had received more education and more were engaged in skilled professions. In addition, there were more younger persons in some geo-political zones that in others. This was confirmed by the statistically significant effect of the sex, educational status, occupation and geopolitical zoning on the tools used for mouth cleaning.

An interesting finding in our study was the fact that many participants using toothpaste were unaware of the fluoride content of their dentrifice. This is less than satisfactory and should be addressed urgently. It is important for people to be able to correctly determine the amount of fluoride they require based on the fluoride content of their water and dentrifices. Although a large number of the dentrifices available in the Nigerian market contain fluoride, there are areas with high levels of fluoride in Nigeria [29] and persons resident in such areas would require lower levels of fluoride in their dentrifices. The lack of knowledge displayed could on one hand result in worse cases of fluorosis in endemic areas or caries in areas where fluoride is inadequate.

A few participants reported using items such as cotton wool, water and salt for mouth cleaning. This is similar to other findings [19, 20, 22], which indicate that a variety of items are used for oral hygiene among Nigerians. There is no scientific evidence on the efficacy of such tools and promotion of their use should be done with caution. The use of other aids such as dental floss and mouthwash was not very common. This is probably because they are not readily available in many parts of the country especially in the rural communities. There is need to increase the availability of these tools as a step towards promoting their use countrywide.

It was surprising to note that more than a third of the participants reported twice-daily mouth cleaning. This is higher than has been observed in previous studies [19–22] but lower that what is reported in other countries [24, 25, 30]. Interestingly, all socio-demographic characteristics examined namely age; educational status and geopolitical zoning were significantly related to frequency of tooth-brushing. Accordingly, we suggest that efforts at promoting good oral hygiene should focus on older persons and persons with less education as they are more likely to have poor oral hygiene practices. A finding also reported in earlier studies [19, 20, 22]. A more sustainable and productive approach may be the promotion of good oral hygiene habits early in life. This is because habits are formed early in life and become difficult to change later in life [31]. This approach is likely to yield better results in the long term. The finding that oral hygiene practices were generally poorer in the northern zones may be unconnected with lower literacy levels in the region [17]. Therefore increased exposure to education may lead to increased oral health awareness in the region.

This study is not without limitations, the major limitation is its dependence on self-reported information; such information is often subject to response bias due to the different interpretations individuals can give to the questions [32]. Also, the responses may have been influenced by the social acceptability of their responses [33]. Another possible source of bias was the fact the survey was conducted in a predetermined location (often the local government headquarters), thus increasing the possibility of excluding persons who are unable to reach the specified location. This was especially noticeable in the south-south geopolitical zone, which comprises many riverine communities accessible only via the waterways. A better approach would have been the conduct of household surveys to reduce bias. The absence of clinical oral health data to compare the impact of perceived and normative oral health status limits the applicability of the results. Despite these limitations, the large sample size as well as the wide national coverage makes this data invaluable and the study provides veritable information on the oral health service utilization and common oral hygiene practices among adult Nigerians as well as socio-demographic factors possibly influencing the observed patterns.

## Conclusion

Presently, most Nigerians assess their oral health status positively and majority report using toothbrushes for maintaining good oral hygiene but display poor dental visiting habits. The study findings suggest that although age, sex, educational status, occupation are important determinants of oral hygiene behavior among Nigerians, other factors may play an important role as well. Further studies to identify other factors influencing oral health behavior are suggested, as the high level of non-attendance demonstrated by the participants is undesirable.

## References

[1] Petersen PE, Kwan S (2004). Evaluation of community-based oral health promotion and oral disease prevention – WHO recommendations for improved evidence in public health practice. Community Dent Health.

[2] US Department of Health and Human Services (2000). Oral health in America: A report of the Surgeon General.

[3] Harford J, Spencer AJ, Slade GD, Spencer AJ, Roberts–Thomson KF (2007). Chapter 7 – Oral Health Perceptions. Australia’s Dental Generations: The National Survey of Adult Oral Health 2004–06.

[4] National Institute for Dental Craniofacial Research (NIDCR): **Chapter 3: Setting Health Priorities, Establishing Oral Health Objectives and Obtaining Baseline Information.** *In Healthy people 2010 Oral health toolkit* Available at: <http://www.nidcr.nih.gov/EducationalResources/DentalHealthProf/HealthyPeople2010/Chapter3.htm>. Accessed on 13 March 2014

[5] Petersen PE (2004). Challenges to improvement of oral health in the 21st century – the approach of the WHO Global Oral Health Programme. Int Dent J.

[6] Frencken JE, Holmgren CJ, Helderman WHP (2002). [Online]. Basic package of oral care.

[7] Davies RM, Davies GM, Ellwood RP (2003). Prevention. part 4: toothbrushing: what advice should be given to patients?. Brit Dent J.

[8] (FDI) World Dental Federation (2003). Report of the global oral health planning workshop.

[9] Ainamo J, Parviainen K (1979). Occurrence of plaque, gingivitis and caries as related to self- reported frequency of toothbrushing in fluoride areas in Finland. Commun Dent Oral Epidemio;.

[10] Adeniyi AA, Sofola OO, Kalliecharan RV (2012). An appraisal of the oral health system in Nigeria. Int Dent J.

[11] Akpata ES (2004). Oral health in Nigeria. Int Dent J.

[12] Federal Ministry of Health (2012). National Oral Health Policy.

[13] Adegbembo AO, El Nadeef MA (1998). National survey of periodontal status and treatment needs among Nigerians. Int Dent J.

[14] Adair PM, Pine CM, Burnside G, Nicoll AD, Gillett A, Anwar S (2004). Familial and cultural perceptions and beliefs of oral hygiene and dietary practices among ethnically and socio-economically diverse groups. Community Dent Health.

[15] Davidson PL, Rams TE, Andersen RM (1997). Socio-behavioral determinants of oral hygiene practices among USA ethnic and age groups. Adv Dent Res.

[16] Varenne B, Petersen PE, Fourney F, Msellati P, Gary J, Outarra S, Harang M, Salem G (2006). Illness-related behavior and utilization of oral health services among adult city dwellers in Burkina Faso: evidence froma household survey. BMC Health Serv Res.

[17] National Populations Commission (2009). Analytical report at the national level. Population census of the Federal Republic of Nigeria.

[18] Office of Population Censuses and Surveys (OPCS) (1991). Standard Occupational Classification Volume 3.

[19] Sofola OO, Orenuga OO (2005). A survey of the knowledge, attitude and practices of antenatal mothers in Lagos, Nigeria about the primary teeth. Afr J Med Med Sci.

[20] Jeboda SO, Adeniyi AA, Ogunbodede EO (2009). Assessment of preventive oral health knowledge and practices among rural and urban mothers in Lagos state. Niger Postgrad Med J.

[21] Umesi-Koleoso DC, Ayanbadejo PO (2007). Oral hygiene practices among adolescents in Surulere, Lagos State, Nigeria. Nig Q J Hosp Med.

[22] Sofola OO, Agbelusi GA, Jeboda SO (2002). Oral health knowledge, attitude and practices of primary school teachers in Lagos state. Niger J Med.

[23] Adegbembo AO (1994). Household utilization of dental services in Ibadan Nigeria. Comm Dent Oral Epidemiol.

[24] Christensen LB, Petersen PE, Krustrup U, Kjoller M (2003). Self-reported oral hygiene practices among adults in Denmark. Community Dent Health.

[25] Villa A, Kreimer AR, Polimeni A, Cicciù D, Strohmenger L, Gherlone E, Abati S (2012). Self-reported oral hygiene habits among dental patients in Italy. Med Princ Pract.

[26] Christensen LB, Petersen PE, Steding-Jessen M (2007). Consumption of dental services among adults in Denmark 1994–2003. Eur J Oral Sci.

[27] Hjern A, Grindefjord M, Sundberg H, Rosen M (2001). Social inequality in oral health and use of dental care in Sweden. Community Dent Oral Epidemiol.

[28] Jamieson LM, Thomson WM (2006). Adult oral health inequalities described using area-based and household-based socioeconomic status measures. J Public Health Dent.

[29] Akpata ES, Danfillo IS, Otoh EC, Mafeni JO (2009). Geographical mapping of fluoride levels in drinking water sources in Nigeria. Afr Health Sci.

[30] Al-Otaibi M, Zimmerman M, Angmar- Mansson B (2003). Prevailing oral hygiene practices among urban Saudi Arabians in relation to age, gender and socio-economic back- ground. Acta Odontol Scand.

[31] Christensen P (2004). The health-promoting family: a conceptual framework for future research. Soc Sci Med.

[32] Peer E, Gamliel E (2011). Too reliable to be true? response bias as a potential source of inflation in paper-and-pencil questionnaire reliability. Pract Assessment Res Eval.

[33] Fisher RJ, Katz JE (2000). Social‒desirability bias and the validity of self‒reported values. Psychol Mark.

[CR34] The pre-publication history for this paper can be accessed here:http://www.biomedcentral.com/1472-6831/14/140/prepub

